# Longitudinal Analysis of the Deep Brain Stimulation Impairment Scale for Subthalamic Nucleus Stimulation in Parkinson’s Disease

**DOI:** 10.1016/j.prdoa.2025.100354

**Published:** 2025-06-11

**Authors:** Paula Broetzmann, Carolin Semmler, Hannah Jergas, Gregor A. Brandt, Christina van der Linden, Charlotte Schedlich-Teufer, Franziska Maier, Elke Kalbe, Veerle Visser-Vandewalle, Michael T. Barbe, Juan Carlos Baldermann

**Affiliations:** a Department of Neurology, Faculty of Medicine and University Hospital Cologne, University of Cologne, Cologne, Germany; b Department of Psychiatry and Psychotherapy, Faculty of Medicine and University Hospital Cologne, University of Cologne, Cologne, Germany; c Department of Stereotactic and Functional Neurosurgery, Faculty of Medicine and University Hospital Cologne, University of Cologne, Cologne, Germany; d Department of Psychiatry and Psychotherapy, Medical Center – University of Freiburg, Faculty of Medicine, University of Freiburg, Freiburg, Germany

**Keywords:** Deep Brain Stimulation, DBS, STN-DBS, DBS-Impairment Scale, DBS-IS, Quality of life

## Abstract

**Background:**

Deep brain stimulation (DBS) of the subthalamic nucleus (STN) is an established treatment for people with Parkinson’s disease (PwP). However, some patients treated with DBS may still experience quality of life limiting impairments after surgery, e.g., concerning posture and gait, cognition, speech, apathy or impulsivity, as well as technical challenges in managing the implanted device. The Deep Brain Stimulation Impairment Scale (DBS-IS) was developed to assess these impairments, yet their postoperative development is underexplored.

**Methods:**

We retrospectively analysed changes in the DBS-IS Total Score and subscales assessed in patients at baseline, three, six and twelve months post-DBS implantation (n = 33 PwP) using linear mixed models for repeated measurements. Furthermore, we investigated device-related issues within the first year after surgery and their impact on quality of life in a larger sample (n = 99 PwP) using DBS-IS Facility Scores.

**Results:**

After surgery we observed significant improvement in the DBS-IS Total Score (β = −1.93; p = 0.005), particularly in the subscales of postural instability, gait difficulty and cognitive impairment. 43 % of patients reported device-related problems within one year after surgery. Patients with device-related problems showed significantly less improvement in quality of life (z = −2.40; p = 0.017). Exploratory post-hoc tests suggested a higher propensity for device-related complications among older individuals (z = −2.19; p = 0.029).

**Conclusion:**

The majority of impairments evaluated by the DBS-IS, such as postural instability, gait difficulty and cognitive impairments, appear amenable to STN-DBS intervention. Special attention is warranted for potential device issues, especially in older patients, to optimize patients’ quality of life outcomes.

## Highlights

• The DBS-IS was developed to assess quality of life-limiting impairments after surgery, yet their post-surgical development remains unclear.
• Impairments evaluated by the DBS-IS improve after surgery in subscales postural instability and gait difficulty and cognitive impairment.
• Device-related problems are related to reduced quality of life and are more common among older individuals.

## 1 Introduction

Parkinson's disease (PD) is among the most common neurodegenerative disorders that significantly impairs patients' quality of life (QoL) [1,2]. Since the 1990s, subthalamic nucleus (STN) deep brain stimulation (DBS) has been a highly effective treatment for motor symptoms, with a positive impact on, for example, mobility, daily activities, stigma, bodily discomfort and overall QoL [3,4]. In selected patients, STN-DBS is a superior intervention regarding motor complications compared to medical management alone [1]. However, a subset of patients undergoing STN-DBS may experience complications during DBS treatment, such as worsening of speech [5] and communication [4] or no significant improvement in emotional well-being, social support and cognition [3,6]. Increased risk-seeking behaviour [7] and apathy [8] have also been described in patients with STN-DBS. These complications may affect patients' subjective QoL evaluations and can lead to dissatisfactory results of STN-DBS, despite improvements of motor symptoms [9,10]. While motor outcomes following STN-DBS implantation have been extensively studied, factors such as side effect management, handling of the DBS device, and other aspects contributing to quality of life improvements − and how these factors evolve after surgery − require further investigation [11].

In 2016, Maier et al. introduced the DBS-Impairment Scale (DBS-IS, see Supplement 1), a 22-item questionnaire devised to assess typical STN-DBS-related difficulties with the intent to pinpoint causes for patient dissatisfaction in STN-DBS outcomes [12]. The DBS-IS encompasses the following subscales: Postural Instability and Gait Difficulty (PIGD), Cognitive Impairments, Speaking Difficulties, Apathy, Impulsivity, and DBS device-related issues. These factors were identified as critical for QoL after DBS surgery through extensive interviews with patients and caregivers [12]. Importantly, the DBS-IS is not meant to replace the Parkinson Disease Questionnaire − 39 (PDQ-39), commonly used to measure QoL in PwP. Rather, it serves to detect specific symptomatic deficits or maintained impairments experienced during DBS treatment as extracted from interviews with PwP treated with DBS [13]. Haarmann et al. evaluated the DBS-IS as a complement to the PDQ-39 by crossmatching 186 patients with non-DBS counterparts by age, gender, disease onset, occupation, and level of education. In this analysis, the PDQ-39 showed significant group differences in the Mobility and Communication subscales, while the DBS-IS revealed significant differences in PIGD, Speaking Difficulties and Apathy. QoL measured by the PDQ-39 was not significantly lower in patients with DBS, but according to the DBS-IS PIGD and Speaking Difficulties were increased in the DBS group. Thus, Haarmann et al. argued that the DBS-IS in fact measured impairments specifically associated with STN-DBS treatment that are not adequately demonstrated by PDQ-39 [13].

While the cross-sectional relevance of the DBS-IS has been shown, temporal evolution of these DBS-specific impairments in the postoperative period remains unclear. To date, no longitudinal data have been analysed to comprehensively describe the evolution of DBS-specific impairments assessed by the DBS-IS over time. Additionally, no direct comparisons of the pre- and postoperative states were available demonstrating whether impairments observed after DBS surgery were a consequence of the intervention or rather originated from the underlying disease in a specific preselected patient group eligible for DBS (e.g. with early motor fluctuations). Furthermore, individual subscales of the DBS-IS had not been independently analysed over time, leaving a gap in understanding which specific factors are influenced by DBS treatment and which remain unaltered compared to the preoperative status. Conducting such an analysis could be instrumental in accurately characterizing the QoL profile associated with STN-DBS and in distinguishing impairments related to STN-DBS that may hinder improvements in QoL.

Apart from specific symptoms, clinical observation implies that patients‘ well-being can be strongly influenced by issues associated with the DBS device, such as problems with the patient handset or complications associated with the cables and generator. Evidence from other diseases e.g. pneumology, gastroenterology and nephrology indicate a strong correlation between QoL and at-home device issues [14,15]. The precise impact of these device-associated challenges in STN-DBS for PD remained unclear, since systematic investigations of the impact of DBS device management on patients' subjective QoL are rare (e.g. [16]). We aimed to fill this gap by assessing the DBS-IS Facility Score which includes three items: problems with the handset, cables and/or generator, and/or the stimulator in general.

In summary, there was insufficient knowledge about how specific factors that potentially deteriorate QoL in patients after STN-DBS evolve over time after DBS surgery. Therefore, we investigated the course of specific factors derived from the DBS-IS, from the preoperative state up to twelve months after surgery. Our secondary objective was to discern the implications of device-associated complications on QoL improvements after DBS implantation.

## 2 Methods

### 2.1 Study design and participants

In this quantitative retrospective study, we evaluated clinical data and questionnaires. In total, we enrolled 99 patients with idiopathic PD treated with STN-DBS between 2019 and 2022 at the University Hospital of Cologne. All patients had been diagnosed with PD according to MDS clinical diagnostic criteria [17]. There are two samples in our study design. For the first research question concerning longitudinal analysis of the DBS-IS and comparison with baseline values, 33 patients who had completed the DBS-IS questionnaire prior to surgery as well as three, six and twelve months postoperatively were included and are hereafter referred to as ‘Pre-Post DBS cohort’. Additionally, 99 patients were enrolled into our second sample (including those from the Pre-Post DBS cohort). These patients had also been seen for control visits three, six and twelve months postoperatively in our centre; however there was no DBS-IS baseline data collected for the additional 66 patients. This group will be referred to as ‘Post-Operative Facility Score cohort’ and serves to answer the second research question. All patients received bilateral implantation of DBS electrodes in the STN and patients completed the questionnaires under their regular dopaminergic medication (medication-on state) and for the postoperative cohort under their regular stimulation parameters (stimulation-on, medication-on state). Representative stimulation parameters at the three-month postoperative visit for each patient are provided in Supplement 2. A visual reconstruction of the location of active contacts was performed with the Lead-DBS toolbox using the default settings (Version 3.0, www.lead-dbs.org, [18]). The study was approved by the local ethics committee (Number: 24-1082-retro).

### 2.2 Outcome measurements

The DBS-IS questionnaire (see Supplement 1) encompasses six domains that refer to self-reported and thus subjective impairments: Postural Instability and Gait Difficulties (PIGD) (5 items), Cognitive Impairments (5 items), Speaking Difficulties (3 items), Apathy (3 items), Impulsivity (3 items) and DBS device-related difficulties (3 items). Each item is self-rated by the patient from zero (never) to four points (always applies), adding up to the DBS-IS Total Score ranging from 0 to 88 points. The DBS-IS Total Score I-V (i.e. the sum of the subscores PIGD, Cognitive Impairment, Speaking Difficulties, Apathy and Impulsivity) depicts the Total Score without DBS-IS Items 20–22 (the device-related problems) that are only assessed postoperatively and is therefore utilizable in preoperative states. The DBS-IS Facility Score is the sum of DBS-IS Items 20–22 depicting the device-related difficulties: problems with the handset, cables and/or generator, and/or the stimulator in general.

Secondary data was collected to describe the study sample. Demographic variables included gender and age. For the evaluation of QoL, the PDQ-39 was used, which aggregates eight domains into a singular index reflecting subjective health status self-reported by patients [19]. The PDQ-39 Total Score can be calculated as PDQ-39 Summary Index (PDQ-39 SI, ranging from zero to a maximum impairment in score of 100). Motor symptoms were assessed using part three of the Unified Parkinson Disease Rating Scale (UPDRS III) [20], comprising 18 items including tremor, rigidity, bradykinesia and postural instability each ranked from zero to four points (with a maximum score of symptoms in 108). The UPDRS III was assessed off-medication at baseline and at six and twelve month follow-ups in the medication-off/stimulation-on state to estimate the efficacy of STN-DBS on motor symptoms. Additionally, the Montreal Cognitive Assessment (MoCA) [21] was administered by neuropsychologists at each visit to objectively evaluate cognitive function (ranging from 0 to 30, values over 26 generally being considered as normal cognitive function). Additionally, we collected data on medication, summarized as levodopa equivalent daily dose (LEDD) [22], for every patient.

### 2.3 Statistics

For the Pre-Post DBS cohort (n = 33), we analysed mean DBS-IS Total Scores for each timepoint applying a Linear Mixed Effect Model (LME) to test the effect of time on DBS-IS results, including the patient variable as random effect factor (MATLAB syntax: 'fitlme(dbsis_total ∼ timepoint + (1|pat_nr)'). For post-hoc testing we performed non-parametric Wilcoxon signed-rank tests for dependent samples to evaluate outcomes at different timepoints (three, six and twelve months postoperatively) compared to baseline since most variables did not follow a normal distribution, as assessed with the Kolmogorov-Smirnov Test. In a similar way, we evaluated the PDQ-39 SI across all timepoints to illustrate the development of overall QoL. We further employed a similar LME to analyse temporal progression of the DBS-IS subscales, i.e. PIGD, Cognitive Impairment, Speaking Difficulties, Apathy and Impulsivity. To test for multiple comparisons across subscales, we applied a false discovery rate (FDR) adjustment to respective p-values [23]. Post-hoc comparisons with baseline were again performed using Wilcoxon signed-rank tests for dependent samples. To support the representativeness of our sample we also conducted a LME and Wilcoxon signed-rank test for UPDRS-III scores preoperatively and three and twelve months postoperative (collected during the medication-off and medication off/stimulation-on state for the postoperative follow-ups). To further analyse objective cognitive impairment by MoCA in comparison to subjective measurement by DBS-IS Cognitive Impairment subscale, we also conducted a similar LME for temporal progression of MoCA Total Score and corresponding post-hoc test between three, six and twelve months values with baseline (see Supplement 3).

In the Post-Operative Facility Score cohort (n = 99) we explored the proportion of patients experiencing device-related problems as measured by the DBS-IS Facility Score and sought to determine its effect on QoL measured by PDQ-39 SI. Given that the DBS-IS Facility Score is only assessed post-implantation, evaluations were conducted at three, six and twelve months after surgery. To test whether patients experiencing device-related problems differ in QoL from those without problems, we grouped patients into two groups: “Device-Problem” cases (DBS-IS Facility Score > 0 points at least once over the course of one year) and “No Device-Problem” cases (DBS-IS Facility Score = 0). We then compared the percentage change of the PDQ-39 SI from baseline to one year postoperatively between groups using Wilcoxon rank-sum testing. In a final explorative analysis, we tested whether elderly patients and/or patients with higher cognitive impairment would have more device problems as indicated by the DBS-IS Facility Score. To test this, we calculated categorical Wilcoxon rank-sum testing between the groups “Device-Problem” and “No Device-Problem” for age and MoCA Total Score at baseline.

All statistical analyses were performed with a predetermined significance level of p < 0.05 using MATLAB 2021b (The MathWorks Inc., Natick, MA, USA). In general, mean values and standard deviations (SD) are presented.

## 3 Results

### 3.1 Clinical data and demographics

The Pre-Post DBS cohort, in which preoperative DBS-IS assessments were available, comprised 33 patients (12 females and 21 males). The mean age at baseline was 59.85 (±7.50 SD) years. On average, patients had been diagnosed 8.53 (±3.74 SD) years prior to receiving STN-DBS implantation. Motor symptoms, evaluated before surgery by the UPDRS III score in medication-off state, had a mean value of 36.13 (±10.72 SD) points. When assessed in medication-on state, the UPDRS III score averaged 18.78 (±8.19 SD). The baseline LEDD for the sample was 1012 (±355.61 SD) mg.

**Fig. 1:**
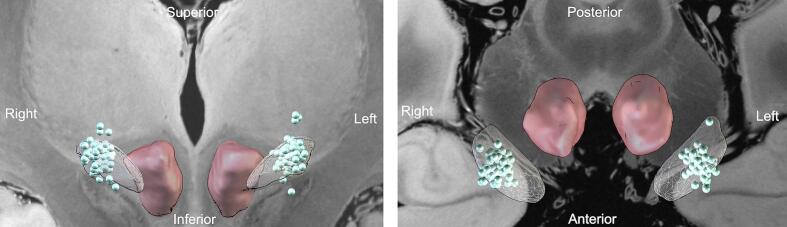
*Location of active contacts in standard space.* For each patient, the active contacts (light blue spheres) used at the three-month follow-up were reconstructed using the Lead-DBS toolbox. The left panel presents a coronal view, while the right panel displays an axial view. The subthalamic nucleus is visualized as a transparent mesh, and the red nucleus is highlighted in red for anatomical orientation. (For interpretation of the references to colour in this figure legend, the reader is referred to the web version of this article.)

The Post-Operative Facility Score cohort comprised 99 patients (36 females and 63 males); all individuals were assessed at three, six and twelve months postoperatively (including all patients from the Pre-Post DBS cohort). The average age at baseline was 62.62 (±7.72 SD) years. Patients had been diagnosed 9.92 (±4.32 SD) years prior to STN-DBS implantation. Motor symptoms, evaluated before surgery using the UPDRS III score in medication-off state, had a mean value of 38.74 (±11.44 SD) and when assessed in medication-on state, the UPDRS III score averaged 20.10 (±8.22 SD). The baseline LEDD for this sample was 1081 (±434.65 SD) mg. All patients were treated with different STN-DBS devices from the companies Medtronic and Boston Scientific (see Table 1). UPDRS III scores improved significantly from the preoperative medication-off state to three and twelve month follow-ups in the medication-off/stimulation-on state (see Table 2). A visualization of the positioning of active contacts from all patients at the three months follow-up is given in Fig. 1.

**Table 1:** Preoperative Clinical and demographic data.

	Pre-Post DBS cohort (n = 33 patients)		Post-Operative Facility Score cohort (n = 99 patients)	
Sex	12 female	21 male	36 female	63 male
Age	59.85 (±7.50)		62.62 (±7.72)	
Disease Duration to STN-DBS	8.53 (±3.74)		9.92 (±4.32)	
UPDRS III at Baseline	Medication-off: 36.13 (±10.72)		Medication-off: 38.74 (±11.44)	
	Medication-on: 18.78 (±8.19)		Medication-on: 20.10 (±8.22)	
LEDD (in mg) at Baseline	1012 (±355.61)		1081 (±434.65)	
Number of Patients with Specific Device	Vercise PC Boston Scientific = 22		Vercise PC Boston Scientific = 51	
	Genus RC Boston Scientific = 7		Genus RC Boston Scientific = 38	
	Percept PC Medtronic = 4		Percept PC Medtronic = 9	
			Genus PC Boston Scientific = 1	

STN-DBS = Subthalamic nucleus deep brain stimulation UPDRS III = Unified Parkinson’s Disease Rating Scale (Part III) LEDD = Levodopa-equivalent daily dosis.

**Table 2:** Longitudinal Analysis of the DBS-IS.

	Baseline (BL)	Change BL to three months	Change BL to six months	Change BL to twelve months	LME		Post-hoc test		
					p	β	BL to three months	BL to six months	BL to twelve months
							p	p	p
DBS-IS Total Score I-V	16.91 (±10.03 SD)	5.04 (±10.10 SD)	6.20 (±9.71 SD)	6.00 (±12.57 SD)	**0.005**	−1.93	**0.029**	**0.013**	**0.020**
− PIGD	6.12 (±4.41 SD)	1.93 (±4.14 SD)	2.05 (±3.04SD)	2.40 (±4.01SD)	0.043*	−0.74	**0.026**	**0.006**	**0.014**
− Cognitive Impairment	4.85 (±3.25 SD)	1.59 (±4.46 SD)	2.29 (±3.54 SD)	1.45 (±2.87 SD)	0.047*	−0.58	**0.039**	**0.016**	**0.030**
− Speaking Difficulties	2.88 (±2.50 SD)	0.82 (±2.94 SD)	1.35 (±2.23 SD)	1.00 (±2.55 SD)	0.085*	−0.33	0.157	0.070	0.097
− Apathy	1.55 (±1.91 SD)	0.57 (±2.04 SD)	0.33 (±1.83 SD)	0.37 (±3.22 SD)	0.209*	−0.03	0.135	0.486	0.414
− Impulsivity	1.52 (±1.77 SD)	0.15 (±2.01 SD)	0.14 (±1.96 SD)	0.60 (±2.11 SD)	0.849*	−0.17	0.650	0.755	0.146
PDQ-39 SI	24.80 (±12.21 SD)	10.27 (±12.57 SD)	11.46 (±14.91 SD)	9.86 (±13.44 SD)	**<0.001**	−3.75	**<0.001**	**<0.001**	**0.001**
UPDRS III	38.49 (±11.65 SD) (med-off)	29.70 (±11.66 SD) (med-off/stim-on)	n.a.	21.73 (±11.65 SD) (med-off/stim-on)	**<0.001**	−8.51	**<0.001**	n.a.	**<0.001**

Given are mean values and standard deviations (SD). Results of timepoints on the respective outcome in a linear mixed effect model (LME) for repeated measures are shown. *FDR corrected p-values.

### 3.2 Longitudinal analysis of the DBS-IS

**Fig. 2:**
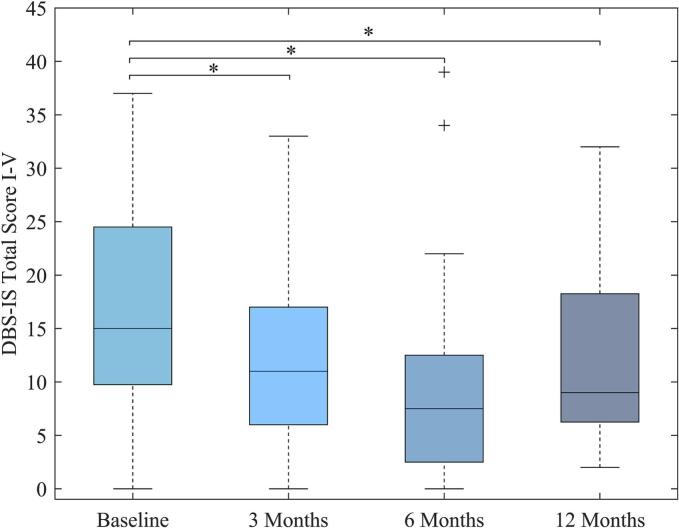
*DBS-IS Total Score Over Time.* Displayed is a boxplot of the DBS-IS Total Score, i.e. the sum of subscales I-V, at each timepoint. The median and corresponding percentiles are represented. Linear mixed effect model revealed a significant change over time. Post-hoc tests indicated a significant improvement from baseline to three, six and twelve months postoperatively (marked with asterisks).

The LME showed a significant effect of time on the DBS-IS Total Score (β = −1.93; p = 0.005, see Fig. 2 and Table 2) in the Pre-Post DBS cohort (n = 33). Post-hoc tests showed a significant improvement from baseline to three months (mean 5.04 points ± 11.52 SD; z = 2.19; p = 0.029), six months (6.20 ± 10.10 SD; z = 2.50; p = 0.013) and twelve months (6.00 ± 9.71 SD; z = 2.33; p = 0.020).

**Fig. 3:**
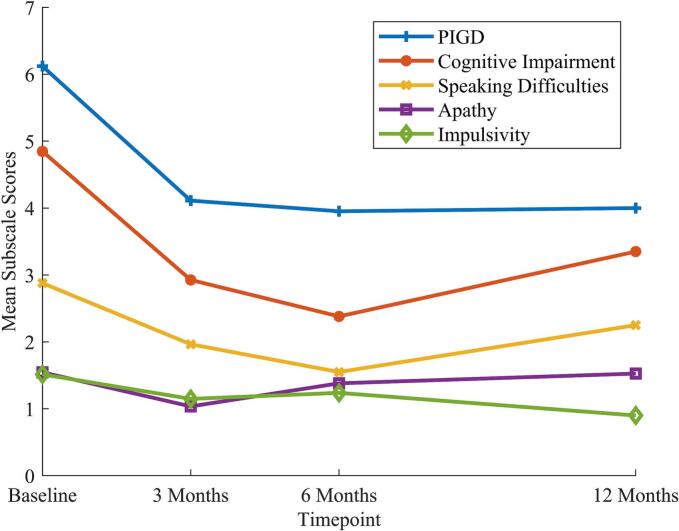
*DBS-IS Total Subscales Over Time.* Displayed is a line plot with mean total scores for each subscale of the DBS-IS at each timepoint. Linear mixed effect model revealed a significant change over time for Postural Instability and Gait Difficulty (PIGD) and Cognitive Impairment subscale while Speaking Difficulties showed a trend. Post-hoc tests indicate significant improvement from baseline to three, six and twelve months postoperatively (see Table 2).

The PIGD subscale also demonstrated a significant effect of time (β = −0.74; p_FDR_ = 0.042). Post-hoc non-parametric Wilcoxon signed-rank tests revealed a significant improvement from baseline to three (1.93 ± 4.14 SD; z = 2.22; p = 0.026), six (2.05 ± 3.04 SD; z = 2.73; p = 0.006) and twelve months (2.40 ± 4.01 SD; z = 2.47; p = 0.014). Additionally, the subscale for Cognitive Impairment had a significant effect of the time (β = −0.59; p_FDR_ = 0.046). Post-hoc testing revealed significant improvement from baseline to three (1.59 ± 4.46 SD; z = 2.06; p = 0.039), six (2.29 ± 3.54 SD; z = 2.41; p = 0.016) and twelve months (1.45 ± 2.87 SD; z = 2.17; p = 0.030). In comparison, the objective measurement of cognition assessed by the MoCA Total Score also improved from baseline to postoperative timepoints but did not show significant effect of time in the calculated LME (see Supplement 3). The subscale for assessing Speaking Difficulties showed a trend for improvement over all timepoints (β = −0.33; p_FDR_ = 0.085) but post-hoc testing revealed no significant change from baseline to three, six and twelve months postoperatively. The subscales for Apathy (β = −0.03; p_FDR_ = 0.209) and Impulsivity (β = −0.17; p_FDR_ = 0.849) did not yield significant results (see Fig. 3 and Table 2).

To illustrate the course of the DBS-IS in comparison to QoL measured by the PDQ-39, we investigated the course of the PDQ-39 SI. We found a significant effect of time on the PDQ-39 SI (β = −3.75; p < 0.001). Post-hoc tests revealed a significant improvement of QoL from baseline to three months (10.27 points; z = 4.77; p < 0.001), six months (11.46 points; z = 3.63; p < 0.001) and twelve months (9.86 points; z = 3.26; p = 0.001, see Table 2).

### 3.3 Analysis of the DBS-IS Facility Score

**Fig. 4:**
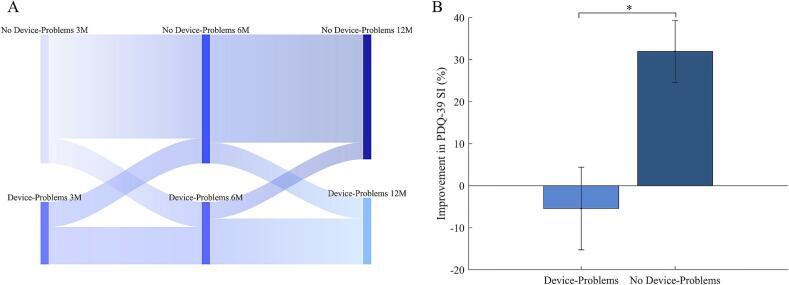
*Distribution of Cases with and without Device-Problems and respective Changes in Quality of Life*, A) The displayed Sankey plot shows the distribution of patients with device-related problems (DBS-IS Facility Score > 0) and without device-related problems (DBS-IS Facility Score = 0) from the total number of patients within the Post-Operative Facility Score cohort at each postoperative timepoint. B) Displayed is the relative improvement of the PDQ-39 SI from baseline to twelve months follow-up for cases with device-related problems (DBS-IS Facility score > 0 in the first year) and cases without. Wilcoxon rank-sum tests reveal a significant difference in PDQ-39 SI changes for both groups (marked with asterisks).

For the analysis of device-related problems we examined the Post-Operative Facility Score cohort (n = 99) after STN-DBS implantation on device-related problems concerning problems with the handset, cables and/or generator, and/or the stimulator in general (DBS-IS Items 20–22). 43 out of 99 patients (43.43 %) had problems with the device at any timepoint within one year after DBS implantation (see Fig. 4a). Patients who reported device-related problems within the first year of DBS showed significantly less improvement in QoL as assessed with the PDQ-39 SI relative change from baseline to 12 months postoperatively (z = −2.40; p = 0.017). Notably patients who reported device-related problems showed a relative decline of 5.42 % (±64.44 SD) in PDQ-39 SI compared to the increase of 31.95 % (±49.90 SD) for patients without reported device-related problems (see Fig. 4b).

**Fig. 5:**
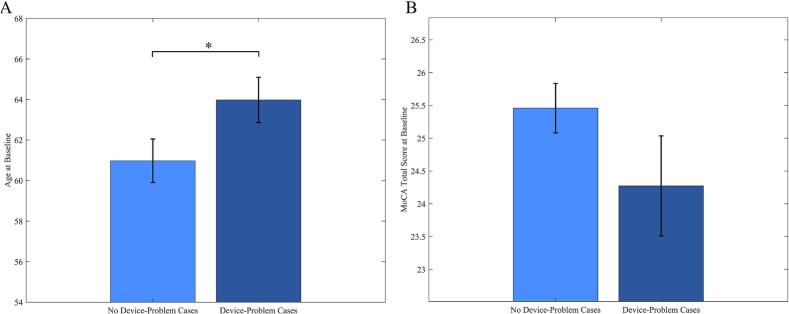
*Baseline Age and Cognitive Impairment measured by MoCA Total Score of Cases with or without Device-Related Problems*, A) Displayed is a bar plot with standard errors for the average baseline age for cases with device-related problems (DBS-IS Facility score > 0 in the first year) and cases without. Wilcoxon rank-sum tests reveal a significant difference baseline age for both groups (marked with asterisks). B) Displayed is a bar plot for averaged baseline MoCA Total Scores for cases with device-related problems (DBS-IS Facility score > 0 in the first year) and cases without.

As potential influencing factors, we tested the impact of age and cognition on the course of the DBS-IS Facility Score within the Post-Operative Facility Score cohort. We identified patient age at baseline as significant determinant for the categorical differentiation between patients with a “Device-Problem” and patients with “No Device-Problem” (mean baseline age of 63.98 ± 7.34 SD years vs. 60.98 ± 7.29 SD years; z = −2.19; p = 0.029) (see Fig. 5a). This indicates that older patients were more likely to have device-related problems. Cognitive impairment objectively measured by MoCA Total Score did not yield significant results as a differentiator between the “Device-Problem” and “No-Device Problem” groups (mean baseline MoCA of 25.46 ± 2.29 SD vs. 24.27 ± 4.38 SD; z = 0.88; p = 0.38) (see Fig. 5b).

## 4 Discussion

In the present study, we used the DBS-IS to characterize the postoperative development of impairments that are typically observed in PwP treated with STN-DBS. Further, we investigated DBS-specific device problems and how they relate to age and cognition. We discovered that overall, impairments assessed by the DBS-IS Total Score improved after surgery over the course of one year. By analysing the different subscales of the DBS-IS separately, we found that this improvement was driven by significant improvements for the subscales PIGD and Cognitive Impairment and with a similar but non-significant trend toward improvement in Speaking Difficulties. Further, we observed that 43.43 % of the patients reported device-related problems within the first year of STN-DBS treatment. Those device-related problems had an impact on overall QoL improvements after DBS implantation. Explorative analysis suggested that device-related problems are related to older age of PwP undergoing STN-DBS.

The DBS-IS is a recently developed questionnaire designed to capture impairments commonly reported by STN-DBS patients [12]. These impairments may limit postoperative QoL improvements, even when motor symptoms improve. In this longitudinal analysis, overall impairments decreased after surgery, notably in the PIGD and Cognitive Impairment domains. Similar improvements in PIGD have been reported using the UPDRS III [24]. In our Pre-Post DBS cohort, self-reported cognitive impairments declined within the first year post-surgery. In contrast, *meta-*analyses report moderate cognitive decline after STN-DBS, though controlled studies attribute this to the natural progression of cognitive dysfunction in PwP [25]. As the DBS-IS captures self-reported rather than objectively measured cognitive changes, it may not align with neuropsychological assessments. One possible explanation for the observed improvements in self-reported cognitive function may be due to the reduction of fluctuations in non-motor symptoms in general, as described by others [26].

In our analysis, Speaking Difficulties did not change significantly after STN-DBS implantation, albeit showing a trend towards improvement. Previous studies had already suggested that vocal outcomes of patients vary significantly [5]. From our own clinical experience, improvements in speaking difficulties that correspond to hypokinetic states, i.e. hypophonia, can be observed in some patients. In fact, kinematic speech analyses show that lip movements improve under DBS [27]. On the other hand, DBS itself can induce side-effects such as dysarthria that results in increased speaking difficulties (see [28] for a detailed discussion of DBS-related changes in speech). Thus, in this regard, a more fine-grained analysis of specific contributors to speaking difficulties may help to inform patients about differential effects on speech.

Self-reported apathy on the DBS-IS remained stable over time, with no significant postoperative changes. This contrasts with two *meta*-analyses reporting increased apathy following STN-DBS, even compared to medication-only treatment [29,30]. However, a recent prospective study of 367 STN-DBS patients found no change in apathy and identified preoperative deficits in action initiation as a predictor of postoperative apathy, indicating a specific vulnerability in certain patients [31]. In our cohort, baseline self-reported apathy was low, and disease duration appeared shorter than in the aforementioned *meta*-analyses. Thus, we speculate that our sample may have been less prone to develop apathy compared to other studies [29,30]. Impulsivity also showed no significant change post-DBS. While many studies report reduced impulsive and compulsive behaviors, likely due to lower dopaminergic medication [29,30], DBS can also induce impulsivity or mania, depending on stimulation spread within the STN [32]. In our preselected sample, consistently low impulsivity scores may have resulted in a statistical floor effect, limiting the detection of changes.

Overall, our results indicate that the impairments, typically observed in patients treated with STN-DBS, tend to generally improve after surgery. Previously, the question has been raised whether the occurrence or severity of the symptoms detected by the DBS-IS could be regarded as side effects of STN-DBS surgery or if they can be regarded as disease-related and may thus represent the severity of the disease in a pre-selected sample, i.e. PwP that qualify for DBS (e.g. because of early motor fluctuations) [33]. In a previous study, a cross-matched analysis of the DBS-IS revealed that PIGD, Speaking Difficulties and Apathy differed in patients with STN-DBS after surgery compared to patients not treated with STN-DBS [13]. Here, a significantly higher impairment in the DBS-IS Total Score was found in the STN-DBS group, with significantly higher impairment regarding PIGD and speaking difficulties. In this study, the control group was matched concerning age and disease duration, but not disease severity in general, e.g., measured with the UPDRSIII in medication-off and stimulation-off status. Thus, it is possible that patients who qualified for DBS had a higher disease severity and a more malignant course of the disease than the control group, despite the comparable disease duration. In line with this, Jahanshahi posited that the DBS-IS might be of limited value in differentiating causality between progression-related and surgically-induced symptoms of PD in the longer term [33]. Indeed, our current data shows that on average, the DBS-IS improves from pre- to postoperative state. This suggests that higher DBS-IS Total scores previously observed in patients with STN-DBS [13] compared to patients without STN-DBS might rather correspond to disease-progression-related than stimulation-induced side effects. Therefore, it is still plausible that some of the patients score higher in the DBS-IS because of stimulation-induced side effects. It is also worth noting that since the development of the DBS-IS, we assume that the field of STN-DBS has advanced when it comes to patient selection, targeting and current shaping [34] which may result in fewer side effects (e.g. in form of dysarthria) in the current sample compared to previous samples, although this remains to be investigated.

We see the additional items summarized in the Facility Score as an important advantage of the DBS-IS questionnaire, being the only validated questionnaire assessing device handling and related problems in DBS patients. In our study, patients who reported technical difficulties in the first year of DBS treatment showed significantly less improvement of QoL after surgery. On average, these patients even showed a slight worsening although the variability was considerably high. It is important to note that our study cannot draw causal conclusions here. It is possible that patients with more malignant forms of PD that may not well respond to DBS are also patients who are more likely to encounter technical difficulties with the device as they report higher motor symptoms and cognitive impairment. On the other hand, issues with device handling, e.g. by accidentally turning the stimulator off or accidentally increasing the stimulation amplitude in an uncontrolled manner may indeed hamper outcomes of DBS. Still, the single items within the Facility Score are rather unspecific. For example, when asking for general problems with the stimulator, we were left with doubt whether patients understood this item as related to the patient programmer, to necessary charging of the device, the impulse generator or other parts of the system. Thus, the issue of device-related problems and their relationship with QoL requires further studies and potentially improved questionnaires that address this topic. On average, patients experiencing device-problems tended to be older than those who did not. Thus, we argue that especially in elderly patients, clinicians should be mindful to address potential problems with device handling and train patients and caregivers accordingly. Eventually, other factors may influence handling of DBS devices. For instance, the way patients are individually trained to use patient programmers certainly may help to prevent device-related issues. On the other hand, individuals living alone with limited access to caregivers and timely support may be at a higher risk of experiencing device-related issues. Another factor that may contribute to device-related issues is the manufacturer of the DBS system and the usability of the patient programmer. In our cohort, over 90 % of patients were treated with devices from the same manufacturer, limiting our ability to determine whether differences in device-related problems could be attributed to variations between manufacturers. Future studies may consider these additional potential confounders when addressing the presence of device-related issues during DBS treatment.

The retrospective nature of this study poses a significant limitation and requires caution in drawing causal inferences. Furthermore, it has to be taken into account that the DBS-IS is a self-reported and therefore subjective score which was not objectively assessed. It is important to note that at the time when the DBS-IS was developed, DBS was reserved for severely affected patients, while the current sample mostly involves patients with tremor dominance or early motor fluctuations. Further, we did not consider individual medication adjustments measured by the LEDD, although we have to acknowledge changes in self-reported impairments e.g. in impulsivity being influenced by medication differences over time. Equally, we did not investigate whether general changes in motor status after DBS may have influenced changes in the DBS-IS. There is therefore also no consideration on stimulation parameters and of reprogramming the DBS regarding self-reported impairments and their effect on the DBS-IS score. All of the above-mentioned factors can significantly influence various aspects of the DBS-IS and give room for further investigations that could not be addressed in this study, due to its naturalistic setting. It should also be acknowledged that the first cohort within our study is rather small (n = 33). As mentioned previously, the DBS-IS Facility Score consists of rather general questions that do not allow us to pinpoint specific causes of device-related issues. Lastly, even though in our centre in Cologne the team includes three special PD nurses involved in the training of patients and caregivers, it is worth highlighting that our conclusions come from monocentric data; other centres may focus even more on patient education in handling the DBS device which may result in fewer device-related problems.

In summary, this real-world study provides valuable insights into clinical outcomes of impairments commonly observed in PwP with STN-DBS. We show that these impairments, as measured by the DBS-IS, still tend to improve due to DBS. Our results indicate that device-related problems are associated with poorer QoL after surgery, especially in older patients. Future research may aim to refine the DBS-IS specifically to explore device-related problems more precisely, investigate predictors of these problems and explore targeted interventions.

## Declaration of Generative AI and AI-assisted technologies in the writing process

During the preparation of this work the authors used OpenAI (2023) ChatGPT (Mar 14 version) in order to improve language and readability. After using this tool/service, the authors reviewed and edited the content as needed and take full responsibility for the content of the publication.

## CRediT authorship contribution statement

**Paula Broetzmann:** Writing – review & editing, Writing – original draft, Visualization, Project administration, Methodology, Investigation, Formal analysis, Data curation, Conceptualization. **Carolin Semmler:** Writing – review & editing. **Hannah Jergas:** Investigation, Data curation. **Gregor A. Brandt:** Writing – review & editing. **Christina van der Linden:** Writing – review & editing. **Charlotte Schedlich-Teufer:** Writing – review & editing. **Franziska Maier:** Writing – review & editing. **Elke Kalbe:** Writing – review & editing. **Veerle Visser-Vandewalle:** Writing – review & editing. **Michael T. Barbe:** Writing – review & editing, Conceptualization. **Juan Carlos Baldermann:** Writing – review & editing, Visualization, Validation, Conceptualization.

## Declaration of competing interest

The authors declare the following financial interests/personal relationships which may be considered as potential competing interests:

P. Brötzmann: reports no disclosures.

C. Semmler: reports no disclosures.

H. Jergas: declares personal fees from Boston Scientific. She received funding from EITHealth and Koeln Fortune.

G. A. Brandt: reports no disclosures.

C. van der Linden: was supported by the Cologne Clinician Scientist Program (CCSP) / Faculty of Medicine / University of Cologne, outside of the submitted work.

C. Schedlich-Teufer: received travel grant from Medtronic.

F. Maier^:^ reports no disclosures.

E. Kalbe: reports no disclosures.

V. Visser-Vandewalle: received financial support for contributions to congresses and advisory boards from Boston Scientific, Medtronic and LivaNova.

M. T. Barbe: received speaker’s honoraria from Medtronic, Boston Scientific, Abbott (formerly St Jude), GE Medical, UCB, Apothekerverband Köln eV, and Bial; research funding from the Felgenhauer-Stiftung, Forschungspool Klinische Studien (University of Cologne), Horizon 2020 (Gondola), Medtronic (ODIS), and Boston Scientific; and advisory honoraria for the Institut für Qualitaet und Wirtschaftlichkeit im Gesundheitswesen.

J. C. Baldermann: reports no disclosures.
