# Supplementary material for: Longitudinal Analysis of the Deep Brain Stimulation Impairment Scale for Subthalamic Nucleus Stimulation in Parkinson’s Disease
Source: Clin Park Relat Disord. 2025 Jun 11;13:100354. doi: 10.1016/j.prdoa.2025.100354 (PMC12206121; doi:10.1016/j.prdoa.2025.100354)
Supplement: Supplementary Data 1 [file mmc1.docx]

**Supplement 1:** 22-item Deep Brain Stimulation – Impairment Scale.

| **The following questions solely refer to your well-being during the last 4 weeks.**  **I, as a patient with Parkinson’s disease, …**  **(never – rarely – sometimes – often – always applies)** | |
| --- | --- |
| Subscales | Items |
| Postural Instability and Gait Difficulties (PIGD) | 1. Had difficulties walking smaller distances (approx. 100m), due to my mobility.  2. Had difficulties going for a walk (approx. 1km), due to my mobility.  3. Had difficulties not to fall whilst walking.  4. Had difficulties keeping my balance.  5. Had difficulties maintaining an upright posture. |
| Cognitive Impairment | 6. Had difficulties concentrating on one topic (e.g. on TV).  7. Had difficulties keeping something in mind, for instance what I had planned to do next.  8. Had difficulties remembering things that date back.  9. Had difficulties finding the right words whilst saying something.  10. Had difficulties staying focused during conversations. |
| Speaking Difficulties | 11. Had difficulties with my speech/ had speech problems.  12. Spoke in such a low voice that people around me could hardly understand me.  13. Had difficulties speaking slowly enough, so people were hardly able to understand me. |
| Apathy | 14. Was uninterested in leaving my flat/house.  15. Needed a lot of effort to do something, even when I was requested to do it.  16. Had no interest in talking to people. |
| Impulsivity | 17. Felt so euphoric and happy without reason that slowing me down was difficult.  18. Made spontaneous decisions without thinking, which I regretted later in some cases.  19. Was so impatient that I wanted to carry out all plans/ideas immediately. |
| Difficulties related to the DBS device | 20. Had difficulties using the hand set.  21. Had difficulties with the generator and/or the cables.  22. Had difficulties handling the brain stimulation device. |

**Supplement 2:** Stimulation parameters for each patient at three months postoperative (I/II)

| PaT  ID | Amp  L | AmP  R | PW  L | PW  R | Freq  L | Freq  R | ChaR  L | ChaR  R | C_1  L | C_2  L | C_3  L | C_4  L | C_5  L | C_6  L | C_7  L | C_8  L | Case  L | C_1  R | C_2 | C_3  R | C_4  R | C_5  R | C_6  R | C_7  R | C_ 8  R | Case  R |
| --- | --- | --- | --- | --- | --- | --- | --- | --- | --- | --- | --- | --- | --- | --- | --- | --- | --- | --- | --- | --- | --- | --- | --- | --- | --- | --- |
| 1 | 2,7 | 1,7 | 50 | 50 | 130 | 130 | 17,55 | 11,05 |  |  |  | -100 |  |  |  |  | 100 |  | -34 | -33 | -33 |  |  |  |  | 100 |
| 2 | 1,7 | 1,7 | 60 | 60 | 130 | 130 | 13,26 | 13,26 |  |  |  |  | -34 | -33 | -33 |  | 100 | -100 |  |  |  |  |  | -100 |  | 100 |
| 3 | 1,5 | 2,5 | 60 | 60 | 130 | 130 | 11,7 | 19,5 |  | -18 | -16 | -16 | -18 | -16 | -16 |  | 100 |  | -14 | -13 | -13 | -20 | -20 | -20 |  | 100 |
| 4 | 1,3 | 1,3 | 60 | 60 | 130 | 130 | 10,14 | 10,14 |  |  |  |  | -34 | -33 | -33 | -60 | 100 |  | -34 | -33 | -33 | -34 | -33 | -33 |  | 100 |
| 5 | 2,1 | 1,6 | 60 | 30 | 130 | 130 | 16,38 | 6,24 |  | -34 | -33 | -33 | -34 | -33 | -33 |  | 100 |  | -34 | -33 | -33 | -34 | -33 | -33 |  | 100 |
| 6 | 1 | 1 | 60 | 60 | 130 | 130 | 7,8 | 7,8 |  |  |  |  | -34 | -33 | -33 |  | 100 |  |  |  |  | -34 | -33 | -33 |  | 100 |
| 7 | 2,1 | 2,1 | 60 | 60 | 130 | 130 | 16,38 | 16,38 |  | -27 | -27 | -26 | -7 | -7 | -6 |  | 100 |  | -27 | -27 | -26 | -7 | -7 | -6 |  | 100 |
| 8 | 1,4 | 1,6 | 60 | 60 | 130 | 130 | 10,92 | 12,48 |  |  |  |  | -33 | -33 | -34 | -60 | 100 |  | -33 | -33 | -34 | -34 | -33 | -33 |  | 100 |
| 9 | 2 | 1,2 | 60 | 60 | 130 | 130 | 15,6 | 9,36 |  | -34 | -33 | -33 |  |  |  |  | 100 |  | -34 | -33 | -33 |  |  |  |  | 100 |
| 10 | 0,5 | 0,5 | 60 | 60 | 130 | 130 | 3,9 | 3,9 |  | -34 | -33 | -33 | -34 | -33 | -33 |  | 100 |  |  |  |  | -34 | -33 | -33 |  | 100 |
| 11 | 3,7 | 2,8 | 60 | 60 | 104 | 104 | 23,09 | 17,47 |  | -15 | -15 | -15 | -15 | -15 | -15 | -10 | 100 |  |  | -45 | -45 |  |  |  | -10 | 100 |
| 12 | 0,9 | 0,7 | 60 | 60 | 130 | 130 | 7,02 | 5,46 |  |  |  |  | -34 | -33 | -33 |  | 100 |  |  |  |  | -34 | -33 | -33 |  | 100 |
| 13 | 1,8 | 2,3 | 60 | 60 | 130 | 130 | 14,04 | 17,94 |  | -34 | -33 | -33 |  |  |  |  | 100 |  | -34 | -33 | -33 |  |  |  |  | 100 |
| 14 | 1,8 | 1,8 | 60 | 60 | 130 | 130 | 14,04 | 14,04 |  |  |  |  | -34 | -33 | -33 |  | 100 |  |  |  |  | -34 | -33 | -33 |  | 100 |
| 15 | 2 | 2,2 | 60 | 60 | 130 | 130 | 15,6 | 17,16 |  | -10 | -10 | -10 | -24 | -23 | -23 |  | 100 |  | -10 | -10 | -10 | -24 | -23 | -23 |  | 100 |
| 16 | 1,9 | 1,4 | 60 | 60 | 130 | 130 | 14,82 | 10,92 |  | -34 | -33 | -33 |  |  | -100 |  | 100 |  | -34 | -33 | -33 | -34 | -33 | -33 |  | 100 |
| 17 | 2,1 | 1 | 40 | 60 | 130 | 130 | 10,92 | 7,8 | -100 |  |  |  |  |  |  |  | 100 |  | -34 | -33 | -33 |  |  |  |  | 100 |
| 18 | 1,4 | 1,4 | 60 | 60 | 130 | 130 | 10,92 | 10,92 |  |  |  |  | -34 | -33 | -33 |  | 100 |  |  |  |  | -34 | -33 | -33 |  | 100 |
| 19 | 0,5 | 0,8 | 60 | 60 | 130 | 130 | 3,9 | 6,24 |  |  |  |  | -34 | -33 | -33 |  | 100 |  |  |  |  | -34 | -33 | -33 |  | 100 |
| 20 | 5,3 | 1,7 | 60 | 50 | 130 | 130 | 41,34 | 11,05 |  |  | -50 |  | -34 | -50 | -33 |  | 100 |  | -34 | -33 | -33 |  |  | -50 | -50 | 100 |
| 21 | 1,2 | 0,9 | 50 | 50 | 130 | 130 | 7,8 | 5,85 |  |  |  |  | -33,33 | -33,33 | -33,33 |  | 100 |  | -23,08 | -23,08 | -30,77 | -25 | -25 | -25 |  | 100 |
| 22 | 1,4 | 1,3 | 60 | 60 | 130 | 130 | 10,92 | 10,14 |  | -34 | -33 | -33 | -34 | -33 | -33 |  | 100 |  |  |  |  | -34 | -33 | -33 |  | 100 |
| 23 | 2,5 | 3 | 50 | 50 | 130 | 130 | 16,25 | 19,5 |  |  |  |  | -34 | -33 | -33 | -100 | 100 |  |  |  |  |  | -100 |  | -100 | 100 |
| 24 | 3,4 | 1,2 | 50 | 50 | 130 | 130 | 22,1 | 7,8 |  |  |  |  | -25 | -25 |  | -100 | 100 |  |  |  |  | -34 | -33 | -33 |  | 100 |
| 25 | 3,3 | 0,8 | 60 | 60 | 130 | 130 | 25,74 | 6,24 |  | -17 | -17 | -16 | -17 | -17 | -16 |  | 100 |  |  |  |  | -34 | -33 | -33 | -40 | 100 |
| 26 | 2,5 | 2,8 | 50 | 50 | 130 | 130 | 16,25 | 18,2 |  | -34 | -33 | -33 | -34 | -33 | -33 |  | 100 |  |  |  |  | -34 | -33 | -33 |  | 100 |
| 27 | 1,2 | 1,2 | 60 | 60 | 130 | 130 | 9,36 | 9,36 |  |  |  |  | -33,33 | -33,33 | -33,33 |  |  |  | -33,33 | -33,33 | -33,33 |  |  |  |  |  |
| 28 | 1,5 | 1,5 | 60 | 60 | 130 | 130 | 11,7 | 11,7 | -60 | -14 | -13 | -13 |  |  |  |  | 100 | -60 | -14 | -13 | -13 |  |  |  |  | 100 |
| 29 | 1,3 | 2,2 | 50 | 50 | 130 | 130 | 8,45 | 14,3 | -100 |  |  |  | -34 | -33 | -33 |  | 100 |  |  |  | -20 |  |  | -80 | -100 | 100 |
| 30 | 2,5 | 1,9 | 60 | 60 | 130 | 130 | 19,5 | 14,82 | -90 | -4 | -3 | -3 |  |  |  |  | 100 | -90 | -4 | -3 | -3 |  |  |  |  | 100 |
| 31 | 3,4 | 3,1 | 60 | 60 | 130 | 130 | 26,52 | 24,18 | -34 | -33 | -33 |  | -25 | -75 |  |  | 100 | -34 | -33 | -33 |  |  |  |  |  | 100 |
| 32 | 1,2 |  | 60 |  | 130 |  | 9,36 |  |  | -34 | -33 | -33 |  |  |  |  | 100 |  |  |  |  |  |  |  |  |  |
| 33 | 1,2 | 3,2 | 60 | 60 | 130 | 130 | 9,36 | 24,96 | -100 |  |  |  | -34 | -33 | -33 |  | 100 | -60 | -14 | -13 | -13 | -34 | -33 | -33 |  | 100 |
| 34 | 0,3 | 2,5 | 60 | 60 | 130 | 130 | 2,34 | 19,5 |  |  |  |  | -33 | -33 | -34 |  | 100 |  |  |  |  | -33 | -33 | -34 |  | 100 |
| 35 | 1,2 | 1,3 | 60 | 60 | 130 | 130 | 9,36 | 10,14 |  |  |  |  | -18 | -16 | -16 | -50 | 100 |  |  | -10 |  | -20 | -20 | -20 | -40 | 100 |
| 36 | 0,7 | 3,2 | 60 | 60 | 130 | 130 | 5,46 | 24,96 |  |  |  |  | -34 | -33 | -33 |  | 100 |  |  |  |  | -34 | -33 | -33 |  | 100 |
| 37 | 2,9 | 2,9 | 60 | 60 | 130 | 130 | 22,62 | 22,62 |  | -34 | -33 | -33 |  |  |  |  | 100 |  |  |  |  | -34 | -33 | -33 |  | 100 |
| 38 | 2,2 | 2,2 | 60 | 60 | 130 | 130 | 17,16 | 17,16 |  | -34 | -33 | -33 | -100 |  |  |  | 100 |  | -34 | -33 | -33 |  |  | -100 |  | 100 |
| 39 | 1,8 | 1,8 | 60 | 60 | 130 | 130 | 14,04 | 14,04 |  |  |  |  | -34 | -33 | -33 | -50 | 100 |  |  |  |  | -34 | -33 | -33 | -50 | 100 |
| 40 | 1 | 0,6 | 60 | 60 | 130 | 130 | 7,8 | 4,68 |  | -34 | -33 | -33 | -34 | -33 | -33 |  | 100 |  | -21 | -21 | -20 | -13 | -13 | -12 | -30 | 100 |
| 41 | 2 | 2 | 60 | 60 | 130 | 130 | 15,6 | 15,6 |  | -34 | -33 | -33 | -18 | -16 | -16 |  | 100 |  | -34 | -33 | -33 | -18 | -16 | -16 |  | 100 |
| 42 | 0,8 | 1,8 | 60 | 60 | 60 | 60 | 2,88 | 6,48 | -30 | -50 | -35 | -35 | -33 | -50 |  |  | 100 | -60 | -20 | -20 | -20 | -34 | -33 | -33 |  | 100 |
| 43 | 2,2 | 0,9 | 90 | 90 | 130 | 130 | 25,74 | 10,53 |  | -18 | -16 | -16 | -16 | -16 | -16 |  | 100 |  |  |  |  | -34 | -33 | -33 |  | 100 |
| 44 | 1,1 | 1,2 | 60 | 60 | 130 | 130 | 8,58 | 9,36 |  |  |  |  | -34 | -33 | -33 |  | 100 |  |  |  |  | -34 | -33 | -33 |  | 100 |
| 45 | 1,2 | 1,5 | 50 | 60 | 130 | 130 | 7,8 | 11,7 | -100 |  |  |  |  |  |  |  | 100 | -125 | -100 |  |  |  |  |  |  | 100 |
| 46 | 0,5 | 2,2 | 60 | 60 | 130 | 130 | 3,9 | 17,16 |  |  |  |  | -34 | -33 | -33 |  | 100 |  |  |  | -100 |  |  |  |  | 100 |
| 47 | 1,5 | 1,7 | 60 | 60 | 130 | 130 | 11,7 | 13,26 | -50 | -34 | -33 | -33 |  |  |  |  | 100 |  | -34 | -33 | -33 | -18 | -16 | -16 | -50 | 100 |
| 48 | 0,8 | 0,9 | 60 | 60 | 130 | 130 | 6,24 | 7,02 |  | -34 | -33 | -33 | -42 | -42 | -16 |  | 100 |  | -34 | -33 | -33 | -31 | -42 | -27 |  | 100 |
| 49 | 0,8 | 0,8 | 90 | 60 | 130 | 130 | 9,36 | 6,24 |  | -34 | -33 | -33 |  |  |  |  | 100 |  | -34 | -33 | -33 |  |  |  |  | 100 |
| 50 | 0,6 | 1,5 | 50 | 50 | 130 | 130 | 3,9 | 9,75 |  | -33,33 | -33,33 | -33,33 | -33,33 | -33,33 | -33,33 |  | 100 |  | -83,33 | -83,33 | -83,33 | -33,33 | -33,33 | -33,33 |  | 100 |
| 51 | 1,6 | 1,2 | 60 | 60 | 130 | 130 | 12,48 | 9,36 |  |  |  |  | -34 | -33 | -33 |  | 100 |  |  |  |  | -34 | -33 | -33 |  | 100 |
| 52 | 2,1 | 1,8 | 60 | 60 | 130 | 130 | 16,38 | 14,04 |  | -34 | -33 | -33 | -50 | -50 |  |  | 100 |  | -34 | -33 | -33 | -100 |  |  |  | 100 |
| 53 | 1,6 | 1,4 | 60 | 60 | 130 | 130 | 12,48 | 10,92 |  |  |  |  | -34 | -33 | -33 |  | 100 |  | -10 | -10 | -10 | -34 | -33 | -33 |  | 100 |
| 54 | 2 | 0,5 | 90 | 60 | 159 | 159 | 28,62 | 4,77 |  | -34 | -33 | -33 |  |  | -40 | -60 | 100 |  |  |  |  | -34 | -33 | -33 |  | 100 |
| 55 | 2,3 | 2,5 | 60 | 60 | 159 | 159 | 21,94 | 23,85 |  | -34 | -33 | -33 |  |  |  |  | 100 |  | -34 | -33 | -33 |  |  |  |  | 100 |
| 56 | 0,9 | 0,9 | 60 | 60 | 130 | 130 | 7,02 | 7,02 |  |  |  |  | -34 | -33 | -33 |  | 100 |  |  |  |  | -34 | -33 | -33 |  | 100 |
| 57 | 0,8 | 1 | 60 | 60 | 130 | 130 | 6,24 | 7,8 |  |  |  |  |  |  |  |  |  |  |  |  |  |  |  |  |  |  |

**Supplement 2:** Stimulation parameters for each patient at three months postoperative (II/II)

| PaT  ID | Amp  L | AmP  R | PW  L | PW  R | Freq  L | Freq  R | ChaR  L | ChaR  R | C_1  L | C_2  L | C_3  L | C_4  L | C_5  L | C_6  L | C_7  L | C_8  L | Case  L | C_1  R | C_2 | C_3  R | C_4  R | C_5  R | C_6  R | C_7  R | C_ 8  R | Case  R |
| --- | --- | --- | --- | --- | --- | --- | --- | --- | --- | --- | --- | --- | --- | --- | --- | --- | --- | --- | --- | --- | --- | --- | --- | --- | --- | --- |
| 58 | 2,5 | 2,2 | 50 | 50 | 130 | 130 | 16,25 | 14,3 |  |  |  |  | -34 | -33 | -33 |  | 100 |  | -34 | -33 | -33 |  |  |  |  | 100 |
| 59 | 2,3 | 1,2 | 60 | 60 | 130 | 130 | 17,94 | 9,36 |  |  |  |  | -2 | -10 | -8 | -90 | 100 |  |  |  |  | -34 | -33 | -33 |  | 100 |
| 60 | 0,5 | 0,5 | 60 | 60 | 130 | 130 | 3,9 | 3,9 |  | -34 | -33 | -33 |  |  |  |  | 100 |  |  |  |  | -34 | -33 | -33 |  | 100 |
| 61 | 1,6 | 1,2 | 60 | 60 | 130 | 130 | 12,48 | 9,36 |  |  |  |  | -27 | -27 | -26 | -20 | 100 |  |  |  |  | -27 | -27 | -26 | -20 | 100 |
| 62 | 0,2 | 0,2 | 60 | 60 | 130 | 130 | 1,56 | 1,56 |  |  |  |  | -34 | -33 | -33 |  | 100 |  |  |  |  | -34 | -33 | -33 |  | 100 |
| 63 | 2,5 | 2,4 | 60 | 60 | 130 | 130 | 19,5 | 18,72 |  |  |  |  | -34 | -33 | -33 |  | 100 |  |  |  |  | -34 | -33 | -33 |  | 100 |
| 64 | 0,8 | 0,8 | 90 | 90 | 130 | 130 | 9,36 | 9,36 |  |  |  |  |  |  |  | -100 | 100 |  |  |  |  | -14 | -13 | -13 | -60 | 100 |
| 65 | 2,1 | 2,6 | 60 | 60 | 130 | 130 | 16,38 | 20,28 |  |  |  |  | -34 | -33 | -33 |  | 100 |  |  |  |  | -18 | -100 | -16 | -50 | 100 |
| 66 | 2 | 2 | 60 | 60 | 130 | 130 | 15,6 | 15,6 |  |  |  |  | -34 | -33 | -33 |  | 100 |  |  |  |  | -34 | -33 | -33 |  | 100 |
| 67 | 1 | 2 | 60 | 60 | 130 | 130 | 7,8 | 15,6 |  |  |  |  | -34 | -33 | -33 |  | 100 |  |  |  |  | -34 | -33 | -33 |  | 100 |
| 68 | 1,2 | 1,2 | 60 | 60 | 130 | 130 | 9,36 | 9,36 |  |  |  |  | -33,33 | -33,33 | -33,33 | -33 | 100 |  | -34 | -33 | -33 | -33,33 | -33,33 | -33,33 |  | 100 |
| 69 | 4,4 | 3,1 | 50 | 50 | 130 | 130 | 28,6 | 20,15 |  |  |  |  | -34 | -33 | -33 |  | 100 |  | -34 | -33 | -33 |  |  |  |  | 100 |
| 70 | 0,4 | 0,4 | 60 | 60 | 130 | 130 | 3,12 | 3,12 |  | -34 | -33 | -33 |  |  |  |  | 100 |  | -34 | -33 | -33 |  |  |  |  | 100 |
| 71 | 1,4 | 1 | 60 | 60 | 130 | 130 | 10,92 | 7,8 |  | -34 | -33 | -33 |  |  |  |  | 100 |  | -34 | -33 | -33 |  |  |  |  | 100 |
| 72 | 0,9 | 1,1 | 60 | 60 | 130 | 130 | 7,02 | 8,58 |  | -34 | -33 | -33 | -20 | -15 | -15 | -50 | 100 |  | -27 | -27 | -26 | -7 | -7 | -6 | -50 | 100 |
| 73 | 2,5 | 2,5 | 60 | 60 | 130 | 130 | 19,5 | 19,5 |  | -34 | -33 | -33 | -34 | -33 | -33 |  | 100 |  | -34 | -33 | -33 |  |  |  |  | 100 |
| 74 | 3,7 | 2,4 | 60 | 60 | 130 | 130 | 28,86 | 18,72 |  |  |  |  | -34 | -33 | -33 |  | 100 |  | -34 | -33 | -33 | -20 | -20 | -20 |  | 100 |
| 75 | 2 | 2 | 60 | 60 | 130 | 130 | 15,6 | 15,6 |  |  |  |  | -34 | -33 | -33 |  | 100 |  | -34 | -33 | -33 |  |  |  |  | 100 |
| 76 | 2,5 | 2,8 | 50 | 50 | 130 | 130 | 16,25 | 18,2 |  | -34 | -33 | -33 |  |  |  | -30 | 100 |  |  |  |  | -34 | -33 | -33 | -30 | 100 |
| 77 | 1,3 | 0,6 | 60 | 60 | 130 | 130 | 10,14 | 4,68 |  | -34 | -33 | -33 | -34 | -33 | -33 |  | 100 |  | -34 | -33 | -33 | -34 | -33 | -33 |  | 100 |
| 78 | 3,4 | 3,6 | 40 | 40 | 130 | 130 | 17,68 | 18,72 |  |  |  |  | -33 | -34 | -33 |  | 100 |  |  |  |  | -33 | -34 | -33 |  | 100 |
| 79 | 1,5 | 0,3 | 60 | 60 | 130 | 130 | 11,7 | 2,34 |  |  |  |  | -34 | -33 | -33 |  | 100 |  |  |  |  | -34 | -33 | -33 |  | 100 |
| 80 | 2,4 | 2,3 | 60 | 60 | 130 | 130 | 18,72 | 17,94 |  | -17 | -17 | -17 | -17 | -16 | -16 |  | 100 |  | -17 | -17 | -17 | -17 | -16 | -16 |  | 100 |
| 81 | 2,2 | 2,1 | 60 | 60 | 130 | 130 | 17,16 | 16,38 |  | -34 | -33 | -33 | -34 | -33 | -33 |  | 100 |  | -34 | -33 | -33 |  | -33 | -33 |  | 100 |
| 82 |  |  |  |  |  |  |  |  |  |  |  |  |  |  |  |  |  |  |  |  |  |  |  |  |  |  |
| 83 | 1,2 | 1,3 | 600 | 60 | 130 | 130 | 93,6 | 10,14 |  | -34 | -33 | -33 |  |  |  |  | 100 |  | -34 | -33 | -33 |  |  |  |  | 100 |
| 84 | 0,8 | 0,8 | 60 | 60 | 130 | 130 | 6,24 | 6,24 |  |  |  |  | -33 | -33 | -34 |  | 100 |  |  |  |  | -33 | -33 | -34 |  | 100 |
| 85 |  |  |  |  |  |  |  |  |  |  |  |  |  |  |  |  |  |  |  |  |  |  |  |  |  |  |
| 86 | 2 | 2,3 | 60 | 60 | 130 | 130 | 15,6 | 17,94 |  | -34 | -33 | -33 | -20 | -20 | -20 | -40 | 100 |  | -34 | -33 | -33 | -30 | -30 | -30 | -10 | 100 |
| 87 | 2,5 | 3,5 | 60 | 60 | 130 | 130 | 19,5 | 27,3 |  | -34 | -33 | -33 | -34 | -33 | -33 |  | 100 |  | -34 | -33 | -33 | -50 | -50 |  |  | 100 |
| 88 |  |  |  |  |  |  |  |  |  |  |  |  |  |  |  |  |  |  |  |  |  |  |  |  |  |  |
| 89 |  |  |  |  |  |  |  |  |  |  |  |  |  |  |  |  |  |  |  |  |  |  |  |  |  |  |
| 90 | 2 | 2 | 50 | 50 | 130 | 130 | 13 | 13 | -100 | -34 | -33 | -33 |  |  |  |  | 100 |  |  |  |  | -34 | -33 | -33 |  | 100 |
| 91 |  |  |  |  |  |  |  |  |  |  |  |  |  |  |  |  |  |  |  |  |  |  |  |  |  |  |
| 92 | 3,5 | 1,5 | 60 | 60 | 130 | 130 | 27,3 | 11,7 |  |  |  |  | -34 | -33 | -33 |  | 100 |  |  | -100 |  | -34 | -33 | -33 |  | 100 |
| 93 | 1,5 | 1,5 | 60 | 60 | 130 | 130 | 11,7 | 11,7 | -20 | -34 | -33 | -33 |  | -100 |  |  | 100 |  | -34 | -33 | -33 | -14 | -13 | -13 |  | 100 |
| 94 | 1,5 | 1,6 | 50 | 50 | 130 | 130 | 9,75 | 10,4 |  |  |  |  | -33,33 | -33,33 | -33,33 |  | 100 |  | -40 | -33,33 | -33,33 |  |  |  |  | 100 |
| 95 | 1,6 | 1,2 | 60 | 60 | 130 | 130 | 12,48 | 9,36 |  | -34 | -33 | -33 |  |  |  |  | 100 |  | -34 | -33 | -33 |  |  |  |  | 100 |
| 96 | 2,8 | 0,6 | 60 | 60 | 130 | 130 | 21,84 | 4,68 | -100 |  | -50 |  |  |  |  |  | 100 | -100 |  |  |  | -34 | -33 | -33 |  | 100 |
| 97 | 1,8 | 1,5 | 60 | 60 | 130 | 130 | 14,04 | 11,7 |  |  |  |  | -33,33 | -33,33 | -33,33 |  | 100 |  |  |  |  | -27,78 | -27,78 | -27,78 |  | 100 |
| 98 | 1,6 | 1,7 | 60 | 60 | 130 | 130 | 12,48 | 13,26 |  | -50 | -50 |  | -34 | -33 | -33 |  | 100 |  |  |  |  | -34 | -33 | -33 |  | 100 |
| 99 | 1,4 | 2,1 | 60 | 60 | 130 | 130 | 10,92 | 16,38 |  | -8 | -6 | -6 | -28 | -26 | -26 |  | 100 |  | -8 | -6 | -6 | -28 | -26 | -26 |  | 100 |
| L = Left, R = Right, Amp = Amplitude, PW = Pulse Width, Freq = Frequency, CHARG = Charged, C1-8 = Contacts | | | | | | | | | | | | | | | | | | | | | | | | | | |

**Supplement 3:** MoCA Total Score – Analysis of temporal progression.

To illustrate the course of the DBS-IS Cognition Score in comparison to cognition measured objectively by the MoCA, we additionally investigated temporal progression of the MoCA Total Score. We found improvement but non-significant effect of time on the MoCA Total Score (β = 1.972; p = 0.193) in the calculated LME. Post-hoc tests revealed a significant improvement of QoL from baseline to three months (1.14 points ± 2.47 SD; z = 4.77; p = 0.043) but non-significant improvement to six months (0.25 points ± 2.89 SD; z = 3.63; p = 0.734) and twelve months (1.05 points ± 3.30 SD; z = 3.26; p = 0.192) (see Supplement 2 – Figure 1).


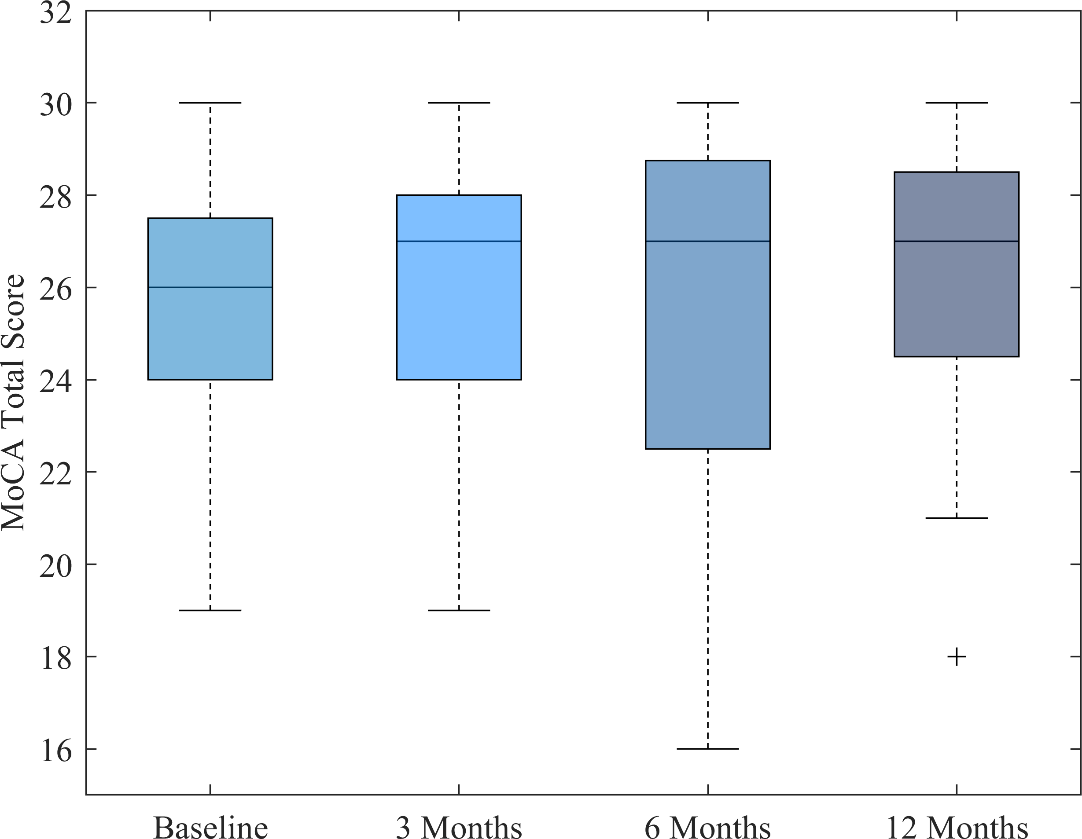


*

Supplement 3 - Figure 1: MoCA Total Score Over Time. *Displayed is a boxplot of the MoCA Total Score at each timepoint. The median and corresponding are represented. Linear mixed effect model revealed no significant change over time. Wilcoxon rank-sum tests reveal a significant difference in MoCA Total Score from baseline to three months (marked with asterisks) but non-significant differences to six and twelve months.*
